# 4-[4-(4-Amino-1,2,5-oxadiazol-3-yl)-1,2,5-oxadiazol-3-yl]-1,2,5-oxadiazol-3-amine

**DOI:** 10.1107/S1600536812017825

**Published:** 2012-04-28

**Authors:** Si-Yuan Jia, Bo-Zhou Wang, Xue-Zhong Fan, Ping Li, Seik Weng Ng

**Affiliations:** a Xi’an Modern Chemistry Research Institute, Xi’an 710065, People’s Republic of China; b Department of Chemistry, Jining Teachers College, Wulanchabu 012000, Inner Mongolia, People’s Republic of China; c Department of Chemistry, University of Malaya, 50603 Kuala Lumpur, Malaysia; d Chemistry Department, King Abdulaziz University, PO Box 80203 Jeddah, Saudi Arabia

## Abstract

The complete molecule of the compound, C_6_H_4_N_8_O_3_, is generated by a crystallographic twofold rotation axis that runs through the central ring. The flanking ring is twisted by 20.2 (1)° with respect to the central ring. One of the amino H atoms forms an intra­molecular N—H⋯N hydrogen bond; adjacent mol­ecules are linked by N—H⋯N hydrogen bonds forming a chain running along [10-2].

## Related literature

For the synthesis, see: Kulikov & Kakhova (1994 ▶); Zhou *et al.* (2007 ▶).
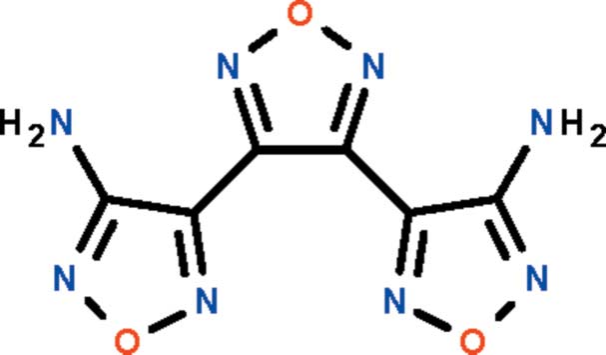


## Experimental

### 

#### Crystal data

C_6_H_4_N_8_O_3_
*M* *_r_* = 236.17
Monoclinic, 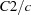

*a* = 7.1681 (9) Å
*b* = 10.8147 (13) Å
*c* = 12.3448 (18) Å
β = 103.155 (1)°
*V* = 931.9 (2) Å^3^
*Z* = 4
Mo *K*α radiation
μ = 0.14 mm^−1^
*T* = 293 K
0.33 × 0.26 × 0.17 mm

#### Data collection

Bruker SMART APEX diffractometer
2675 measured reflections
1047 independent reflections
933 reflections with *I* > 2σ(*I*)
*R*_int_ = 0.014

#### Refinement

*R*[*F* ^2^ > 2σ(*F* ^2^)] = 0.031
*wR*(*F* ^2^) = 0.096
*S* = 1.08
1047 reflections
87 parameters
All H-atom parameters refined
Δρ_max_ = 0.28 e Å^−3^
Δρ_min_ = −0.17 e Å^−3^

### 

Data collection: *APEX2* (Bruker, 2009 ▶); cell refinement: *SAINT* (Bruker, 2009 ▶); data reduction: *SAINT*; program(s) used to solve structure: *SHELXS97* (Sheldrick, 2008 ▶); program(s) used to refine structure: *SHELXL97* (Sheldrick, 2008 ▶); molecular graphics: *X-SEED* (Barbour, 2001 ▶); software used to prepare material for publication: *publCIF* (Westrip, 2010 ▶).

## Supplementary Material

Crystal structure: contains datablock(s) global, I. DOI: 10.1107/S1600536812017825/bt5881sup1.cif

Structure factors: contains datablock(s) I. DOI: 10.1107/S1600536812017825/bt5881Isup2.hkl

Supplementary material file. DOI: 10.1107/S1600536812017825/bt5881Isup3.cml

Additional supplementary materials:  crystallographic information; 3D view; checkCIF report

## Figures and Tables

**Table 1:** Hydrogen-bond geometry (Å, °)

*D*—H⋯*A*	*D*—H	H⋯*A*	*D*⋯*A*	*D*—H⋯*A*
N4—H1⋯N1	0.90 (2)	2.37 (2)	2.932 (2)	121 (1)
N4—H2⋯N3^i^	0.87 (2)	2.23 (2)	3.070 (2)	162 (2)

Symmetry code: (i) 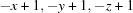.

**Fig. 1.**
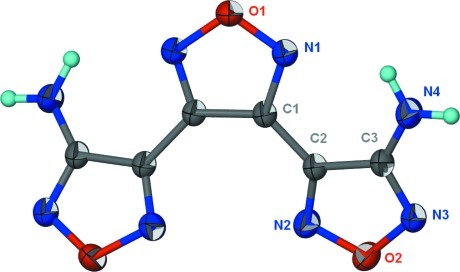
Anisotropic displacement ellipsoid plot (Barbour, 2001) of C6H4N8O3 at the 50% probability level; hydrogen atoms are drawn as spheres of arbitrary radius. The molecule is located on a twofold rotation axis; symmetry-related atoms are not labeled.

**Fig. 2.**
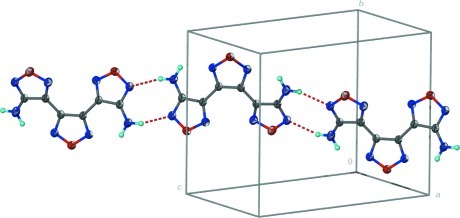
Hydrogen-bonded chain structure. The intermolecular H bond is drawn as a dashed line, the intramolecular H bond is not shown.

## supplementary crystallographic information

### Comment

We are interested in *N*-heterocyclic compounds having few hydrogen atoms as these compounds are a source of explosives. In the title compound (Scheme I), the hydrogen atoms constitute an amino group. In NH_2_–C_2_N_2_O–C_2_N_2_O–C_2_N_2_O–NH_2_, two amino-subsituted 1,2,5-oxadiazole rings flanking a central 1,2,5-oxadiazole ring; the molecule lies on a twofold rotation axis that relates one flanking ring to the other (Fig. 1). The flanking ring is twisted by 20.2 (1) ° with respect to the central ring. One of the amino H atoms forms an intramolecular hydrogen bond; adjacent molecules are linked by an N–H···N hydrogen bond (Table 1, Fig. 2). to form a chain running along [1 0 -2].

### Experimental

3,4-Bis(4'-aminofurazano-3')furoxan was synthesized by using a literature procedure (Zhou *et al.*, 2007). The compound (7.5 g) was dissolved in acetic acid (30 ml). The solution was added to a reducing agent prepared from stannous chloride dihydrate (22.6 g. 100 mm mol) dissolved in acetic anhydride (20 ml), acetic acid (100 ml) and concentrated hydrochloric acid (20 ml). The reduction was performed according to an literature procedure (Kulikov & Kakhova, 1994). The mixture was heated atto 348 K for 8 h. The cool mixture was then poured into water (150 ml). The white precipitate that separated was collected and recrystallized from an ethyl acetate/ether mixture; yield 70%, m.pt. 456–457 K. The purity was established by HPLC to be 99.6%. CH&N elemental analysis. Calculated for C_6_H_4_N_8_O_3_ (%): C 30.51, N 47.46, H1.69. Found: C 30.41, N 47.58,H 1.61.

### Refinement

The H-atoms were located in a difference Fourier map, and were refined freely.

### Crystal data

**Table:**

C_6_H_4_N_8_O_3_	*F*(000) = 480
*M**_r_* = 236.17	*D*_x_ = 1.683 Mg m^−^^3^
Monoclinic, *C*2/*c*	Mo *K*α radiation, λ = 0.71073 Å
Hall symbol: -C 2yc	Cell parameters from 1575 reflections
*a* = 7.1681 (9) Å	θ = 3.4–27.7°
*b* = 10.8147 (13) Å	µ = 0.14 mm^−^^1^
*c* = 12.3448 (18) Å	*T* = 293 K
β = 103.155 (1)°	Prism, colorless
*V* = 931.9 (2) Å^3^	0.33 × 0.26 × 0.17 mm
*Z* = 4	

### Data collection

**Table:**

Bruker SMART APEX diffractometer	933 reflections with *I* > 2σ(*I*)
Radiation source: fine-focus sealed tube	*R*_int_ = 0.014
Graphite monochromator	θ_max_ = 27.5°, θ_min_ = 3.4°
ω scans	*h* = −9→9
2675 measured reflections	*k* = −14→13
1047 independent reflections	*l* = −15→8

### Refinement

**Table:**

Refinement on *F*^2^	Secondary atom site location: difference Fourier map
Least-squares matrix: full	Hydrogen site location: inferred from neighbouring sites
*R*[*F*^2^ > 2σ(*F*^2^)] = 0.031	All H-atom parameters refined
*wR*(*F*^2^) = 0.096	*w* = 1/[σ^2^(*F*_o_^2^) + (0.0566*P*)^2^ + 0.219*P*] where *P* = (*F*_o_^2^ + 2*F*_c_^2^)/3
*S* = 1.08	(Δ/σ)_max_ < 0.001
1047 reflections	Δρ_max_ = 0.28 e Å^−^^3^
87 parameters	Δρ_min_ = −0.17 e Å^−^^3^
0 restraints	Extinction correction: *SHELXL97* (Sheldrick, 2008), Fc^*^=kFc[1+0.001xFc^2^λ^3^/sin(2θ)]^-1/4^
Primary atom site location: structure-invariant direct methods	Extinction coefficient: 0.020 (3)

### Fractional atomic coordinates and isotropic or equivalent isotropic displacement parameters (Å^2^)

**Table:**

	*x*	*y*	*z*	*U*_iso_*/*U*_eq_	
O1	0.0000	0.80892 (10)	0.7500	0.0471 (3)	
O2	0.16242 (12)	0.35531 (8)	0.56878 (8)	0.0482 (3)	
N1	0.09563 (14)	0.73719 (9)	0.68904 (8)	0.0431 (3)	
N2	0.06415 (14)	0.41475 (9)	0.63634 (9)	0.0446 (3)	
N3	0.29963 (15)	0.43342 (9)	0.54042 (9)	0.0453 (3)	
N4	0.3974 (2)	0.63687 (11)	0.58898 (12)	0.0626 (4)	
H1	0.372 (2)	0.7101 (14)	0.6165 (12)	0.059 (4)*	
H2	0.480 (3)	0.6342 (15)	0.5474 (15)	0.064 (5)*	
C1	0.06039 (15)	0.62259 (9)	0.71097 (9)	0.0349 (3)	
C2	0.13553 (15)	0.52528 (10)	0.65104 (9)	0.0358 (3)	
C3	0.28563 (16)	0.53785 (10)	0.59138 (10)	0.0393 (3)	

### Atomic displacement parameters (Å^2^)

**Table:**

	*U*^11^	*U*^22^	*U*^33^	*U*^12^	*U*^13^	*U*^23^
O1	0.0644 (8)	0.0324 (6)	0.0520 (7)	0.000	0.0291 (6)	0.000
O2	0.0540 (5)	0.0385 (5)	0.0600 (6)	−0.0051 (4)	0.0291 (4)	−0.0103 (4)
N1	0.0529 (6)	0.0356 (5)	0.0471 (6)	−0.0024 (4)	0.0248 (5)	−0.0011 (4)
N2	0.0460 (5)	0.0397 (5)	0.0549 (6)	−0.0046 (4)	0.0255 (5)	−0.0068 (4)
N3	0.0513 (6)	0.0401 (5)	0.0526 (6)	0.0004 (4)	0.0287 (5)	−0.0004 (4)
N4	0.0745 (8)	0.0423 (6)	0.0915 (10)	−0.0118 (5)	0.0614 (8)	−0.0088 (6)
C1	0.0364 (5)	0.0344 (5)	0.0373 (5)	−0.0010 (4)	0.0151 (4)	0.0007 (4)
C2	0.0374 (5)	0.0349 (6)	0.0390 (6)	−0.0004 (4)	0.0166 (4)	0.0010 (4)
C3	0.0435 (6)	0.0366 (6)	0.0436 (6)	0.0017 (4)	0.0223 (5)	0.0022 (4)

### Geometric parameters (Å, º)

**Table:**

O1—N1^i^	1.3685 (11)	N4—C3	1.3419 (16)
O1—N1	1.3686 (11)	N4—H1	0.896 (16)
O2—N2	1.3684 (12)	N4—H2	0.871 (19)
O2—N3	1.4001 (13)	C1—C1^i^	1.434 (2)
N1—C1	1.3055 (14)	C1—C2	1.4582 (15)
N2—C2	1.2967 (15)	C2—C3	1.4419 (15)
N3—C3	1.3077 (15)		
			
N1^i^—O1—N1	110.94 (11)	N1—C1—C2	117.95 (9)
N2—O2—N3	110.93 (8)	C1^i^—C1—C2	133.62 (6)
C1—N1—O1	106.22 (9)	N2—C2—C3	109.39 (10)
C2—N2—O2	106.07 (9)	N2—C2—C1	123.83 (9)
C3—N3—O2	105.40 (9)	C3—C2—C1	126.57 (10)
C3—N4—H1	121.5 (10)	N3—C3—N4	124.54 (11)
C3—N4—H2	118.5 (11)	N3—C3—C2	108.19 (10)
H1—N4—H2	118.6 (15)	N4—C3—C2	127.25 (11)
N1—C1—C1^i^	108.31 (6)		
			
N1^i^—O1—N1—C1	0.18 (6)	N1—C1—C2—C3	17.47 (17)
N3—O2—N2—C2	−0.32 (13)	C1^i^—C1—C2—C3	−167.05 (15)
N2—O2—N3—C3	0.77 (13)	O2—N3—C3—N4	177.65 (12)
O1—N1—C1—C1^i^	−0.43 (14)	O2—N3—C3—C2	−0.87 (13)
O1—N1—C1—C2	176.12 (8)	N2—C2—C3—N3	0.73 (14)
O2—N2—C2—C3	−0.23 (13)	C1—C2—C3—N3	−174.16 (11)
O2—N2—C2—C1	174.83 (10)	N2—C2—C3—N4	−177.73 (13)
N1—C1—C2—N2	−156.72 (11)	C1—C2—C3—N4	7.4 (2)
C1^i^—C1—C2—N2	18.8 (2)		

Symmetry code: (i) −*x*, *y*, −*z*+3/2.

### Hydrogen-bond geometry (Å, º)

**Table:**

*D*—H···*A*	*D*—H	H···*A*	*D*···*A*	*D*—H···*A*
N4—H1···N1	0.90 (2)	2.37 (2)	2.932 (2)	121 (1)
N4—H2···N3^ii^	0.87 (2)	2.23 (2)	3.070 (2)	162 (2)

Symmetry code: (ii) −*x*+1, −*y*+1, −*z*+1.
